# Stretching on chromosomes sheds light on their architecture

**DOI:** 10.1038/s41392-022-01141-5

**Published:** 2022-08-23

**Authors:** Ramona Jühlen, Wolfram Antonin

**Affiliations:** Institute of Biochemistry and Molecular Cell Biology, Medical School, RWTH Aachen University, Aachen, Germany

**Keywords:** Cell biology, Biophysics

In a recent study published in Nature, Meijering et al.^1^ employ elegant optical trapping and manipulation experiments of mitotic chromosomes to gain insights into their biomechanical behavior. During mitosis, chromosomes rearrange into iconic X-shaped bodies comprising two rod-shaped sister chromatids connected at the centromere. How mitotic chromosomes condense and adopt their characteristic form has been highly controversial but recent ideas mostly converge into an overarching model.^2^ The new experiments by Meijering et al.^1^ support most of the recent concepts about chromosome structure and indicate how to rigorously test these.

During mitosis, replicated chromosomes condense and rearrange into compact cylindrical bodies to allow their faithful segregation so that each daughter cell collects one copy of the genome. This process, crucial for perpetuation of the hereditary information and cellular and organismic health, requires individualization of chromosomes into separate units, longitudinal shortening of the chromosome axis, overall volume reduction of the chromatin, and resolution of sister chromatids. Decades of research employing light and electron microscopy as well as more recently Hi-C sequencing techniques have generated different models of how mitotic chromosomes acquire their typical shape describing them as a linear array of bead-like granules, coiled filaments, brush-like assembly of DNA-loops or a mass of disordered chromatin.^2,3^ The function of factors identified as crucial for mitotic chromosome formation, typically referred to as chromatin condensation factors, has been controversial and confusing. Depending on the experimental system the results were undecided whether the factors were needed for chromatin compaction, formation of the rod-like structure of mitotic chromosomes or rather their maintenance. Only recently, models converge to the idea that each chromatid within a mitotic chromosome is arranged around a central organizing proteinaceous scaffold, long highly controversial, with consecutive loops of chromatin arranged in a helical staircase configuration. Here, DNA topoisomerase IIα and KIF4A are seen as central components of the scaffold axis. Condensins, two circular protein complexes, which as the name implies were originally identified as chromatin condensation factors, actively form the chromatin loops. The two condensin complexes seem to have distinct but also overlapping functions.

**Fig. 1:**
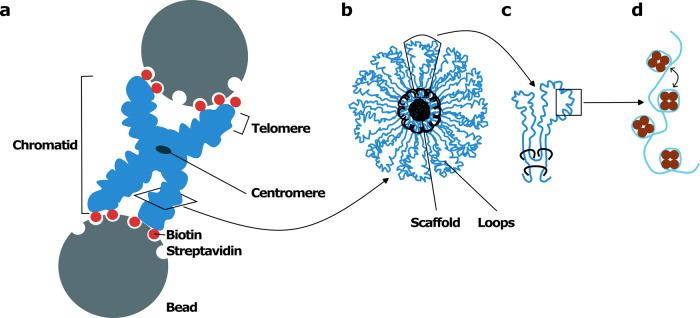
Scheme of force measurements on a mitotic chromosome using optical tweezers manipulation and model of chromosome organization. **a** In the optical tweezer setup a single chromosome, consisting of two chromatids, is trapped between two streptavidin-coated beads by interaction with their biotinylated telomeres, the end points of each chromosome arm. Pulling the two beads apart reveals mechanical properties of the chromosome. **b** Scheme of a cross section through a chromosome arm with the central scaffold indicated in black and looped-arranged chromatin in blue. **c** Chromatin loops (blue) with condensin I and II complexes (black). **d** Magnified chromatin region indicating the nucleosome arrangement with histone octamers (brown) around which the DNA-stand (light blue) wraps. Further compaction possibly arises due to histone modifications favoring inter-nucleosome interaction indicated by the two-headed arrow

Meijering et al. now establish an elegant biophysical assay using optical tweezers, where highly focused laser beams hold micron-sized objects in a microfluidic flow cell and apply forces to them, to study the mechanical properties of native human mitotic chromosomes.^1^ Mitotic chromosomes are isolated from tissue culture cells carrying a fusion of a biotin ligase (BirA) to a protein (TRF1) specifically found at telomeres, the ends of chromosomes. This allows attaching the isolated chromosomes via their biotinylated telomeric ends to streptavidin-coated microspheres (Fig. 1a). If a chromosome gets trapped between two microspheres, the optical tweezer can move these beads and press and pull on the chromosome. In this setup, the force applied can be precisely determined and the chromosome’s resistance to the force as well. By this, mechanical features of the chromosomes can be characterized with very high resolution under controlled conditions.

When pulling the authors find that chromosomes are relatively soft for small extensions. However, when further extended, chromosomes show a pronounced non-linear stiffening, i.e., the stiffness of the chromosomes increases with the force, which needs to be applied to further stretch the chromosomes. This behavior is inconsistent with classical models describing homogeneous polymer network mechanics, which usually reflects well the mechanical properties of architecture-dependent biological functions like for cytoskeletal elements. It rather points to an intrinsic heterogeneity of the mitotic chromosomes’ mechanical properties. The authors thus develop a new, so-called hierarchical worm-like chain model for the chromosome’s mechanical behavior. It describes the chromosome as consisting of many heterogeneous elements or modes that successively stiffen. The heterogeneity might indicate that different regions within the chromatids, like telomeres, centromeres, where the two chromatids are connected, but also regions enriched for eu- or heterochromatin along the chromatid arms might respond differently to tension (Fig. 1a), which might be revealed by innovative Hi-C sequencing approaches. Alternatively, uneven distribution of chromatin condensation factors along the chromatid axis, as indicated by immunostainings,^2^ might account for this.

The strategy also allows investigating the role of specific proteins. In the current study, the authors found that upon depletion of topoisomerase IIα the chromosomes display rather unchanged stiffening behavior. However, when perturbing the chromosomes’ structure by swelling them with high salt concentrations and then relaxing them back to their original shape, control chromosomes did not show strong changes in their stiffness, but chromosomes lacking topoisomerase IIα did. This is consistent with the idea that topoisomerase IIα plays a role in maintaining chromosome architecture without being strictly required for establishing it.^4^

Using this strategy, it will be interesting to test the impact of other factors implicated in mitotic chromosomes’ structure and maintenance such as condensins, cohesins or KIF4A as well as the effect of changes in posttranslational histone modifications such as deacetylation (Fig. 1b–d), which have been suggested to drive mitotic chromatin compaction.^5^ Changes in chromosome numbers as well as chromosome defects are the most common cause of spontaneous abortions but are also implicated in diseases like cancer. The presented innovation will help defining whether changes in chromosomes’ mechanical behavior underlie these pathologies and, if so, might even pave way to read out these changes for diagnostics.
